# Multidimensional Family-Centred Early Intervention in Children with Hearing Loss: A Conceptual Model

**DOI:** 10.3390/jcm11061548

**Published:** 2022-03-11

**Authors:** Daniel Holzinger, Johannes Hofer, Magdalena Dall, Johannes Fellinger

**Affiliations:** 1 Research Institute for Developmental Medicine, Johannes Kepler University Linz, 4020 Linz, Austria; johannes.hofer@bblinz.at (J.H.); magdalena.dall@jku.at (M.D.); johannes.fellinger@bblinz.at (J.F.); 2 Institute of Neurology of Senses and Language, Hospital of St. John of God, 4020 Linz, Austria; 3 Institute of Linguistics, University of Graz, 8010 Graz, Austria; 4 Department of Paediatrics I, Innsbruck Medical University, 6020 Innsbruck, Austria; 5 Division of Social Psychiatry, University Clinic for Psychiatry and Psychotherapy, Medical University of Vienna, 1090 Vienna, Austria

**Keywords:** hearing loss, deaf, early intervention, family-centred, conceptual model, working mechanism, intervention effects

## Abstract

At least two per thousand newborns are affected by hearing loss, with up to 40% with an additional disability. Early identification by universal newborn hearing screening and early intervention services are available in many countries around the world, with limited data on their effectiveness and a lack of knowledge about specific intervention-related determinants of child and family outcomes. This concept paper aimed to better understand the mechanisms by which multi-dimensional family-centred early intervention influences child outcomes, through parent behaviour, targeted by intervention by a review of the literature, primarily in the field of childhood hearing loss, supplemented by research findings on physiological and atypical child development. We present a conceptual model of influences of multi-disciplinary family-centred early intervention on family coping/functioning and parent–child interaction, with effects on child psycho-social and cognitive outcomes. Social communication and language skills are postulated as mediators between parent–child interaction and non-verbal child outcomes. Multi-disciplinary networks of professionals trained in family-centred practice and the evaluation of existing services, with respect to best practice guidelines for family-centred early intervention, are recommended. There is a need for longitudinal epidemiological studies, including specific intervention measures, family behaviours and multidimensional child outcomes.

## 1. Introduction

### 1.1. Children with Hearing Loss

Disabling hearing loss (HL), defined by a hearing threshold above 30 decibels in the better hearing ear, globally affects around 34 million children below the age of 15 [1]. In the Western neonatal population, HL shows a prevalence of about two per thousand, with about two-thirds of the newborns with a bilateral and one-third with a unilateral loss [2]. However, the majority of children with HL live in low- and middle-income countries, with prevalence rates almost four times higher in South Asia, Asia Pacific and Sub-Saharan Africa [3]. Children with HL are a highly diverse group, related to the severity, laterality (bi- or unilateral) and type of HL, their nonverbal cognitive functioning or additional disabilities affecting up to 40% of them [4,5]. About 70% of congenital HL is of genetic origin. Of those with genetic aetiology, about one-third is syndromic [6].

Universal newborn hearing screening programmes, early fitting with hearing aids and/or cochlear implantation and early access to modern early intervention programmes have improved outcomes in children with HL. Positive effects of early intervention have particularly been demonstrated for language development [7,8,9,10]. Nevertheless, a large proportion of children with HL still lag behind their peers with typical hearing. A recent North American multi-centre study on early language outcomes in children with bilateral HL, reported mean expressive vocabulary outcomes about 1.5 standard deviations below average, even after excluding children with additional disabilities, and decreasing vocabulary scores as chronological age increased [11]. Other studies on children with hearing aids or cochlear implants confirm an average language gap of 1–2 standard deviations as compared to children with typical hearing [12,13,14]. Beyond difficulties in the development of structural language skills, the social use of language (pragmatics) has been described as a major persisting area of concern [15,16,17]. A systematic review of the literature demonstrated that children and adolescents with HL were more prone to developing psychopathology (depression, aggression, oppositional defiant disorder, conduct disorder) than their normally hearing peers [18]. In contrast, a more recent study on social development in children with early cochlear implants and typical cognitive functioning did not find significant differences in terms of psychosocial development, as compared to their peers with typical hearing, except with regard to prosocial behaviour [19]. For reading, that is highly correlated with school performance, significant delays have been described in children with HL [20]. Only about half of the teenagers with cochlear implants attain an age-appropriate level of reading comprehension [21]. In contrast to risk for academic underachievement later in life, a recent study on 385 pre-schoolers with HL showed that those who started receiving early intervention by the age of 6 months had similar rates of kindergarten readiness to the total population of kindergarteners [22]. A substantial number of studies report cognitive delays in children with HL, particularly elevated risks for delays in social cognition [23,24] and executive functioning [25,26,27]. 

### 1.2. Families with Children with HL

Child development results from dynamic interactions of the child, with his or her family and in a wider social context. To understand the development of a child with HL, consideration of his/her family context is imperative. Family systems are constantly evolving, including families with one or more primary caregivers, blended families and extended family members, such as grandparents [28]. Families with children with HL are highly diverse. About 95% of the children with HL have parents with typical hearing and, therefore, usually with no experience of HL [29]. As in the general population, families vary with regard to material resources (socio-economic-status), and personal parental resources (parental education, problem-solving ability, mental and physical health, parenting skills, coping styles), the family’s availability of social networks, migration background and family language. 

### 1.3. Early Intervention

According to the WHO, over 60% of global HL could be avoided through preventive measures [1] and, as compared to other childhood developmental disorders, HL and its impact on child development have been shown to be highly malleable by intervention measures. Early intervention is a system of “services and supports that are available to babies and young children with developmental delays and their families”. For the field of HL, best practice documents [30,31,32] refer to a combination of *medical–audiological intervention* and the family’s involvement in a *family-centred early intervention* programme that makes intervention effective. The medical–audiological component includes the early identification of HL through universal newborn hearing screening, followed by effective tracking systems, high-quality otorhinolaryngological, audiological and developmental paediatric diagnostics, and provision of optimal amplification (such as hearing aids or implant technologies), as well as medical guidance over the years. 

The concept “family-centred” was adopted from health care that included families in the treatment of children with special needs in the 1960s. Bronfenbrenner [33] first used the term family-centred in early intervention, to refer to a shift from a focus on child-centred approaches to working with and supporting their families [34]. Family-centred practice has since then been widely used and elaborated and substantiated over the years. Support that involves the families who usually spend most of the time with the child is more powerful than exclusively child-centred intervention [35]. However, it still is not uncommon among medical professionals to assume that the early provision with hearing devices alone will ensure optimal child outcomes. 

Early intervention usually refers to services up to the age of three years, before the child transitions to pre-school. In some countries, early intervention programmes for children with sensory impairments are available up to school entry. Globally, early intervention services often are not specialised for children with HL. In most parts of the world, early intervention for children with HL includes otorhinolaryngological services, developmental paediatric services (particularly for children with additional handicaps), audiological services (hearing assessment and fitting of hearing devices) and (mostly) family-centred early intervention programmes, with the aim to build capacities of the family. In addition, therapeutic services (such as speech–language or occupational therapy), psychological counselling or parent-organized self-help organizations contribute to intervention. 

The development of our conceptual model on influences of early intervention, on children with HL and their families, is based on a general model of early childhood intervention and parent-implemented models of language interventions. Guralnick’s developmental systems model is composed of three different levels, with the aim of demonstrating mechanisms that impact child development [36]. The first level of *Child Development* includes developmental resources (e.g., cognition, language, motor, social–emotional) and organizational processes (e.g., executive function, social cognition, motivation, emotion regulation) that constitute the basis for a child’s social and cognitive competence. The second level is *Family Patterns of Interaction* with three types of family patterns (parent–child transactions, family-orchestrated child experiences and health and safety provided by the family) through which parents influence children’s development. The third level is *Family Resources*, which enable parents to adjust to the children’s characteristics and needs, including personal parent characteristics (e.g., mental and physical health, problem solving ability, coping styles and perceived competence and confidence, attitude and cognitive readiness), material resources (financial resources) and social support. Effective early childhood intervention concentrates on all three levels, with a focus on optimal family patterns of interaction, including parent–child interaction. Another well-established model that specifically addresses the manner in which family systems intervention practices are related to parent–child interaction and child development is the family systems intervention model by Trivette, Dunst and Hamby [37,38]. The four components of the model include help-giving practices, family concerns and priorities, family strengths, and social supports and resources. Following this model, practitioners use help-giving practices so that family members identify their needs and resources, use their strengths and develop new abilities to obtain the necessary supports and resources to meet the family’s needs. The system is based on Bronfenbrenner’s [39,40] postulation that parents can only interact with their children in a way that enhances their development if they are provided with the necessary supports to have the time and energy for their parental responsibilities. Parent-implemented language interventions for children with, or at risk for, language impairment strengthen parent skills for promoting child language development in everyday parent–child interactions [41]. Language learning was shown to be most efficient when children actively engaged with adults who contingently responded to the child’s verbal and nonverbal signals, and provided linguistic modelling, adapted to the child’s current needs [42,43]. Parent-implemented language interventions are implemented in shared book reading, play and home routines [44,45]. 

## 2. Aims and Methods

In this paper we aim to develop a comprehensive conceptual model of influences of non-medical and medical early intervention on children with HL and their families. All the included influences on child development are supported by empirical evidence mostly from the field of HL, supplemented by general child development research. In addition to evidence from the literature the model is based on experiences of the authors who have been providing family-centred early intervention (FCEI) and clinical multi-professional support well connected with network partners (e.g., otorhinolaryngology, hearing aid acousticians, parent associations) over a long period of time. Regarding the references to the literature, we favoured longitudinal multicentre studies if available. 

The model obviously cannot include all relevant mechanisms for child development, and in many cases directions of influences are not exclusively unidirectional. We rather intended to depict the most relevant influences of effective early intervention specifically on children with HL. Having referred to the diversity of children with HL and most relevant developmental outcomes in the introduction, we first discuss the impact of a child’s HL on the family system. After that, the model’s central working mechanism of parent–child interaction is dealt with. The main component of the model concerns direct and indirect effects of family-centred intervention on parent–child interaction and child development, and in the end, on family quality of life. 

**Figure 1:**
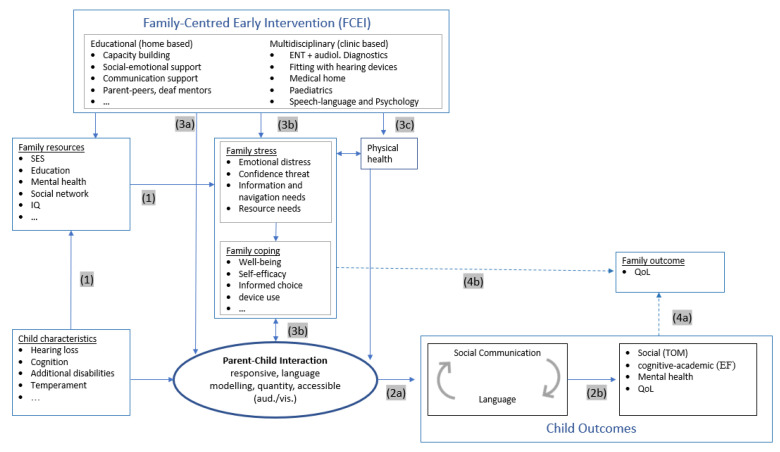
Conceptual model of working mechanisms influencing the relationship between childhood hearing loss and developmental outcomes. EF: executive function; QoL: quality of life; SES: socioeconomic status; ToM: Theory of Mind.

Specifically, we aim to demonstrate (Figure 1):

1. Effects of a child’s HL on the family (1);
2. Effects of parent–child interaction on child language/communication (2a) and more distal domains of child development (2b);
3. Effects of Early Intervention on parent–child interaction and child developmental outcomes:
  - Family-centred education early intervention (3a) and
  - Multidisciplinary clinical intervention (3b).
4. Effects of Early intervention on family quality of life (4).

## 3. Conceptual Model

Figure 1 shows the conceptual model of early intervention in children with HL. Throughout the text, each pathway from the model is referred to by a number (1–4), highlighted in grey.

### 3.1. Effects of a Child’s HL on the Family (1)

#### 3.1.1. Family Emotional Distress

The diagnosis of a child’s HL is a major challenge for his or her family. The negative emotions of parents (mainly mothers), such as shock, denial, guilt, anger and depression are reported by several studies [46,47]. These phenomena are not specific to the diagnosis of HL but general human reactions to unexpected bad news, which impose significant stress on parents of children with any kind of health challenge [48]. Before the introduction of neonatal screening, parents reported frustration about delays in the diagnostic process [49]. Since neonatal screening has been implemented, parents still report emotional reactions, including shock and grief; however, a study by Young and Tattersal [50] suggests that parental distress is moderated by the benefits of early detection and early intervention. A qualitative analysis of parental experiences, with early detection of HL, does not only describe the abovementioned stress-related reactions, but also the need for emotional support in the diagnostic process [46].

Within the coping process, several stages of defences can occur [48]. Besides other phenomena, such as shock, anger, depression and feelings of guilt, denial can be a specific reaction to a primarily invisible condition, such as HL in a newborn [51]. Denial becomes apparent, not only through the parents’ refusal to accept the diagnosis, but, for instance, through missing appointments, which delay further evaluation and intervention. Lutherman [52] suggested to carefully involve parents in the whole diagnostic process, very directly. Communication difficulties between parents and professionals are often reported [46]. Problems in handling the provided information [53], the lack of coordination between different professionals [54], and the need to manage multiple appointments, especially when additional needs of the child have to be met [55], can be a challenge. 

#### 3.1.2. Confidence Threat

Irritation of emotional availability due to the mental state of grief the parents experience, after having received the mostly unexpected diagnosis of HL in their child [56], can be observed, especially in parents with typical hearing [57,58]. Furthermore, the diagnosis of HL may lead to reduced self-confidence in parenting a child with limited access to auditory signals and language, and loss of intuitive parenting [52,59]. This becomes evident in a parenting style that is more directive and less responsive [60,61]. 

#### 3.1.3. Resource Needs

Resource needs connected with the child’s HL, particularly challenges regarding employability [46,62,63,64,65,66], are another stressor on families. 

#### 3.1.4. Navigation and Information Needs

After diagnosis, families usually encounter a variety of medical and non-medical professionals, involved in diagnosis, amplification and intervention. A poor coordination of services is known to be a major cause of ongoing family irritation and a stressor for the family system of children with special health care needs, including those with HL [66,67,68,69]. As one of their primary needs, parents of children with HL expressed the need for specific and clear information [70].

#### 3.1.5. Family Stress over the Course of Time

After the first period of elevated stress levels in coping with the new situation, following the diagnosis of their child’s HL, studies on the extent to which these parents have higher stress levels than parents with children with typical hearing show mixed results [47,63,71,72]. Several studies on general self-reported parenting stress [63,73,74,75] did not report significant group differences, mostly based on investigations using the Parenting Stress Index [76]. Studies based on context-specific instruments, addressing the unique challenge of raising a child with HL and the related communication needs, showed elevated stress levels in hearing families of children with HL [74], which was not found in deaf parents of deaf children [77]. With respect to the course of parental stress levels over time, Lederberg and Golbach (2002) [63] reported increased parental stress in mothers of children with HL, as compared to mothers of children with typical hearing, when their children were 22 months old, but no difference in stress levels at the age of 3–4 years. Heightened stress levels were associated with less satisfaction with social support. Whereas early parental stress is often related to the interaction with health care systems, higher stress levels later in life are often related to educational aspects.

The role of family stress in the proposed model is highly relevant, because increased parental stress has been shown to influence parent–child interactions, contributing to more controlling and less responsive parenting behaviours and less favourable linguistic input [78,79], which is critical for long-term spoken language abilities of children with HL [80,81]. A US study investigated relations between parenting stress, language comprehension and inhibitory control skills, in 39 families of children with HL and 41 families with typically hearing children. No significant differences in levels of general parenting stress or types of stressors (domestic, occupational and financial) were reported. However, in contrast to families with children with typical hearing, greater parenting stress in families with children with HL was associated with poorer spoken language skills in their children, which, in turn, mediated the association with poorer inhibitory control skills [82]. A recent multicentre study, on 164 young children with normal intelligence, with a duration of cochlear implant use of at least three years, focused on possible mechanisms through which parenting stress affects spoken language outcomes and found parental self-efficacy as a mediator to account for 43% of this relationship [83].

Due to the cross-sectional design of the studies, showing significant correlations between parenting stress and child outcomes, little can be said about the direction of this relationship. In line with other studies on child development [37], we expect effects of parental stress on child development. On the other hand, elevated stress levels in parents of children with HL, with poorer language skills or higher rates of behaviour problems [74,75,82,84,85,86], regardless the degree of HL [87], demonstrate that unsatisfactory child outcomes contribute to increased parenting stress. Consequently, a reciprocal (bidirectional) influence between parent–child interaction and family stress is displayed in our model.

### 3.2. Effects of Parent–Child interaction on Child Development (2a)

Before arguing for different pathways of influences of early intervention measures on parent–child interaction and, consequently, on child development (3.3), the role of parent–child interaction in the conceptual model is justified. Parent–child interaction describes the reciprocal, direct relationship between caregiver and child [88]. 

#### 3.2.1. Style of Parent–Child Interaction

Parental responsiveness is the ability to tune in to the child, capturing the child’s current interests and communicative attempts, and the provision of contingent responses. A responsive style of interaction encourages the child’s active communication and, thus, has a positive influence on language development [42]. Parental responsiveness is an aspect of intuitive parenting, where parents naturally adjust their (communicative) behaviours to the child’s needs. However, the lack of a child’s reactions to parental vocalizations can lead to a discouragement of intuitive parenting [61]. Therefore, parents need to learn to tune into their child’s signals (in whatever modality) and try to get the child’s attention in different ways, e.g., by increasing the activity when the child is paying attention and decreasing when the child is looking away [89]. To take over and dominate the conversation can hinder the child from learning interaction strategies. Hearing mothers of children with HL tend to have a more directive communication approach [90], which was, in turn, shown to be linked to lower vocabulary knowledge in children with cochlear implants [91]. One reason for more directive parent behaviour could be that parents assume that their child needs stronger stimulation to encourage reactions [89]. Additionally, parents with typical hearing might not use enough appropriate visual cues and tend to give the child insufficient time to respond [74]. 

Higher maternal sensitivity was associated with better language development, explaining 10% of variance of expressive language gain in a longitudinal study, with 24 children with HL, between 21–30 months at initial testing [92]. In a much larger (*n* = 188) study, with children with cochlear implants, parent–child interaction was investigated, looking at “respect for child autonomy, positive regard, cognitive stimulation, shared visual attention and bi-directional interaction” [93] (p. 1499). It could be shown that, higher scores of parent–child interaction were associated with better growth rates of expressive and receptive language [93]. In the same study cohort, maternal sensitivity and cognitive stimulation positively predicted language growth, even after adjusting for hearing experience and child and other family factors, explaining 11% of the variance in language growth trajectories. Furthermore, there was a significant interaction between maternal sensitivity and linguistic stimulation, meaning that linguistic stimulation only had a positive influence on language growth when maternal sensitivity was high [81]. In addition to the influence of the parenting style on language skills, parenting style has been shown to affect the social–emotional development of the child, with a sensitive, reciprocal and contingent interaction style leading to secure attachment within the first few years of life. An intrusive parenting style can negatively impact later on attachment capabilities [94].

#### 3.2.2. Quantity of Parent–Child Interaction

A recent meta-analysis on the impact of family environment on language development, in children with cochlear implants, demonstrated the impact of parent–child interaction on the language development of children with HL. Both the quantity and quality of parental linguistic input, within the first 4 ½ years of life, explained 31.7% of the variance in child language development [95]. Studies in typically hearing children showed a significant influence of the quantity of parental linguistic input (frequency of words or utterances) directed to a child in their early years on child language development [96,97]. However, more recent studies found that typically hearing children, from middle to upper socio-economic levels, are less susceptible to the amount of their parents’ input when it comes to language learning, whereas children from less advantaged backgrounds and children with HL, especially children using cochlear implants, were dependent on a high amount of interactions with their parents [80,98]. In most studies so far, children with mild to severe HL were exposed to a similar quantity of parental talk at the age of two, as compared to their peers with typical hearing [99,100]. At the age of three, however, it was found that children with HL, even though their parents increased the quality of their linguistic input, were exposed to a smaller total number of words, as well as lower quality of linguistic input, which, in turn, had a negative impact on child language abilities at the age of three [99]. A recent study with children with moderate HL, investigating both the quality and quantity of parental linguistic input in the home environment, concluded that these children received a comparable amount of input as their peers with typical hearing. 

#### 3.2.3. Quality of Parent–Child Communication

The quality of parent–child communication has been shown to have an even higher impact on child language outcomes than the quantity. Parents of children with HL are often less successful in establishing joint attention with their child. A study by Dirks and Rieffe [101] showed the positive relationship between joint attention and language development. Particularly, higher level facilitative language techniques used by parents with their children with HL, such as open-ended questions, recasts (restating the child’s utterance into a question format) or expansions (repeating the child’s utterance by adding new information), enhance child language development, especially during the lexical phase [98,102]. Lower level facilitative language techniques, including imitation, labelling, linguistic mapping and directive language, are beneficial rather for children in the prelexical phase, and hinder language development in the lexical phase [98,103].

In addition to the level of facilitative language techniques, language complexity used by the parents, often measured by the mean length of utterances directed to a child, is associated with receptive and expressive language skills in children with HL [98,104].

#### 3.2.4. Social Communication

Beyond the effects of parent–child interaction on language development, our model illustrates influences of parent–child interaction on the child’s social communication (pragmatic language), i.e., his/her social use of language, the competence to use language as a means of connecting and engaging with others [105]. Through a responsive parental interaction style and the adjustment of communication (e.g., strategies to direct attention, visual communication, pacing of interactions) and language complexity to their child’s needs, within natural social situations, the child does not only learn new words and language structures, but learns how to use them effectively in social interactions, e.g., to attract somebody’s attention, to direct somebody’s attention to an object of interest, to express need for help, to share emotions or to ask for the name of an object by pointing at it. It is within these real demands of social situations that language is acquired. The interrelationship between the acquisition of language and social communication is well supported by Tomasello’s social-pragmatic theory of language learning [106], emphasizing the importance of a child’s understanding of the communicative intentions of others, in a wide variety of interactive situations for the learning of new words, and by social interactionist theories of language learning [107,108]. 

#### 3.2.5. Effects of Social Communication and Language Skills

A large population-based longitudinal study [109] in the UK showed mediating effects of pragmatics, at the age of about 8 years, between early social disadvantage and adolescent mental health outcomes. For children with HL, Ching et al. recently reported, for a sample of 144 9-year-old children with HL, that pragmatic and auditory functional performance, rather than structural language abilities, were associated with better psychosocial abilities and quality of life [110]. In a sample of 33 adolescents, with moderate to profound HL, Zaidman-Zaidman and Most found that pragmatic abilities predicted teacher-reported peer relationship problems and prosocial behaviours, as well as self-reported peer supportive relationships [111]. 

The key role of poor language skills (whether spoken, signed or written), as a consequence of language deprivation from early on, for school success, psycho-social functioning [112,113,114], cognitive development (e.g., social cognition and, specifically, theory of mind [23], executive functioning, such as short term memory or complex problem-solving [27]) and quality of life [115], is based on a rich body of evidence (2b).

### 3.3. Effects of Family-Centred Early Intervention on Parent–Child Interaction and Child Developmental Outcomes

The preceding sections have demonstrated the eminent role of the quality and quantity of parent–child interaction on the social-communication, language and overall development of a child with HL. Furthermore, manifold effects of a child’s HL on the family, such as emotional distress, threats of parental confidence, resource and information/navigation needs, were pointed out in the model so far. 

Pathways of influences of family systems intervention practices on parent–child interactions and development of 910 children, with and without developmental delays, were investigated by meta-analytic structural equation modelling [37]. Family systems intervention showed strong effects on parental self-efficacy beliefs and direct and indirect effects (via self-efficacy beliefs) on parental well-being. In turn, parental-wellbeing was directly related to parent–child interaction and further, to child development. The concept of self-efficacy, grounded in Bandura’s social learning theory [116], is the belief that one is capable of positively influencing child development and confidence, while carrying out parenting tasks to do so.

As a consequence, early intervention needs to take the specific stressors resulting from having a child with HL into account (3b), so that parents regain the energy, motivation and emotional availability required for joyful and effective parent–child interaction. In addition, and due to the eminent role of parent–child interaction for the development of a child with HL, early intervention needs to provide direct parent guidance for optimal adjustment of communication and language to their child’s needs (3a).

#### 3.3.1. Direct Influences of Early Intervention on Parent–Child Interaction

The direct pathway between early intervention and parent–child interaction (3a) represents the coaching and instruction of parents to adapt their verbal and nonverbal interaction to their child and motivating them to exploit the diversity of everyday activities to promote early communication. Implementation of the German Muenster Parental Programme, specifically developed for supporting parents in their very early communication with their infants with HL (3–18 months), showed a significant increase in parent responsiveness to nonverbal and verbal signals of their child, and a reduction in inappropriate initiative parent behaviour, as compared to the control group [117]. In Italy, an adaptation of the ITTT model (it takes two to talk [118,119]), with additional information on HL, hearing devices, hearing environment and listening skills strategies, was implemented in a series of group and a few individual sessions. The programme showed significant effects on the families’ quality of interaction, as compared to a control group, and the children’s language skills increased significantly more in the trained group; the difference was still significant after three years. An Australian study showed the effects of family-focused video interaction guidance on emotional availability (parental sensitivity, non-hostility and responsiveness) and on child involvement, as well as on parent reported self-esteem after intervention [120]. A recent scoping review on interventions on parenting styles of hearing parents of children with HL indicated positive effects of parental, as well as child, communication skills. In addition, parents acquired strengths and coping skills to relate with their child with HL [121]. In a randomized pilot trial, Roberts demonstrated the effects of a parent-implemented communication treatment, delivered in the families’ homes, with a focus on early communication stimulation and parent sensitivity on the frequency of the parents’ use of communication support strategies and children’s gains in speech prelinguistic [122] skills. In line with our proposed model, international consensus on best practice in Family-Centred Early Intervention emphasizes the relevance of consistent language-rich stimulation, during natural interactions with all family members, and accessibility to all communication options [31]. 

#### 3.3.2. Indirect Influence of Early Intervention on Parent–Child Interaction

As a core working mechanism of early intervention, our model shows indirect influences on parent–child interaction via family social and emotional support, helping parents to cope with the stressors related to their child’s HL, and to regain self-efficacy and well-being (3b). Interestingly, lower self-efficacy was reported for parents of children with hearing aids, as compared to parents of children who utilise cochlear implants, which might be related to the amount of support received [123]. Recently, higher levels of self-efficacy, related to higher levels of involvement in family-centred early intervention and higher levels of received support, were reported, specifically, for fathers of children with HL [124,125]. In accordance, the best practice guideline on family-centred early intervention, for children who are deaf or hard of hearing, emphasises FCEI’s role to promote family well-being (e.g., enjoyment of the child, optimism about the child’s future, emotional availability) and self-efficacy (i.e., the families’ competence and confidence in parenting and promoting the child’s development). To achieve this, balanced partnerships between families and professionals and parental informed choice are considered essential. The provision of professional parent–peer support and the connection with other families with children with HL, as a source of social-emotional support and as an informational resource support, are regarded as important by the majority of parents [126,127]. The inclusion of deaf/hard-of-hearing mentors as role models and providers of signed language is another essential element of modern FCEI [30,31,128,129]. When parents connect with deaf adults, they report less feelings of stress and increased confidence about raising a child with HL [130].

#### 3.3.3. Effects of Multidisciplinary Intervention

The following section tries to focus on multidisciplinary clinical intervention and its potential impact on family factors and parent–child interaction, in the context of a multi-dimensional and multi-professional family-centred “medical home”. The American Academy of Paediatrics defined the concept of a medical home for children with special health care needs as an approach to providing health care that is accessible, family-centred, continuous, comprehensive, coordinated, compassionate and culturally competent [131]. Farmer et al. (2011) [132] generally demonstrated a decline in unmet needs, improved satisfaction with specialty care and care coordination, and improved ratings of child health and family functioning for Medical Home concepts, pointing out that effective care coordination seems to be a strong mediator for reduced parenting stress for children with complex needs, including children with HL. Guiding and navigating families of affected children by shared decision-making forms a basic concept of a “medical home” and can support positive coping mechanisms [133].

In our model the medical–audiological intervention is regarded as an essential component of family-centred intervention and not in contrast to the more educational family-centred approach. FCEI does not describe a specific type of intervention (e.g., education, medicine, therapy) but the way interventions are delivered, so that families are supported, informed and empowered and family interactions are strengthened. 

Parents of children with a recent diagnosis of HL are suddenly confronted with a network of professionals, including constant specialist medical, audiological, and other professionals’ appointments, managing hearing aids and/or cochlear implants and face communicative, developmental, parenting and educational challenges. 

There is high need for respectful, family-adjusted navigation and guidance. Therefore, multidisciplinary teams, with the shared goal of delivering the right care at the right time, and in the right place, organising the care coordination around the child’s and the families’ needs are crucial [134,135,136] (3b). According to the Joint Committee on Infant Hearing [30], all infants with confirmed permanent HL should receive early intervention services, as soon as possible after diagnosis, including a simplified, single point of entry into an intervention system that is appropriate for children with HL. The United States Preventative Services Task Force for Hearing Loss recommends family-centred care in the paediatric setting, tailoring the delivery of health services based on parental participation and collaboration with all health professionals who provide healthcare to their child, to reduce family stressors, foster coping mechanisms and ensure the best possible physical health of the child [137].

Close cooperation between home-based early intervention services and hospital-based multidisciplinary services is essential to avoid the irritation of families and to support them in their interaction with their child. In addition to early interventionists, medical doctors and audiologists, parent–peers can have an important function as family navigators and peers in the process of coping with the diagnosis. Social workers and psychological support can help to cope with resource needs and emotional distress.

##### Early Identification of HL

Solid evidence supports the early identification of children with HL and the importance of timely access to family-centred early intervention [138,139]. However, parents describe the pathway from hearing screening to their child’s hearing diagnosis as a stressful and anxious time, strongly interfering with their initial thoughts and hopes around their family’s future, affecting their ability to follow through and manage needs related to their child´s HL [46,56]. Timely, family-centred support and navigation and guidance on the way from hearing screening to HL diagnosis, and along early intervention, is a critical component to foster the best possible long-term outcomes for the child [46,140,141,142]. 

Principles, such as shared decision making and consistent “case management”, are suitable to encounter parents’ needs in this early period. Establishing a multi-professional network, including staff of maternity wards, paediatric units, ENT departments and family-centred intervention providers, is, therefore, advisable [143]. 

##### Device Fitting

Consistent use of hearing devices is crucial for speech and language development in children with HL [93,144,145,146,147,148]. Furthermore, the consistency of audibility throughout the developmental periods significantly impacts speech and language development [149]. 

During the emotional and stressful time of HL diagnosis, parents are challenged by the need for significant decisions regarding their child´s hearing devices and mode of communication [62,150,151]. 

Parents may experience further irritation due to unclear explanations about HL and hearing amplification, and may perceive the received advice as misleading or incorrect, especially when there is inconsistent coordination among the involved professionals [46,152]. 

It is important to consider the influence of the professional information provider on the parent’s decision process and its potential impact on parental stress level and coping mechanisms [151,153,154,155,156]. From the parents’ perspective, there is a “complex relationship between parental need for specifics and straight answers, while insisting on high levels of diplomacy and sensitivity” [70] (p. 2), making a consistent multi-professional intervention team a powerful parent-supporting tool. It is, however, of tremendous importance that the diagnostic and decision-guiding professional teams are aware of the support and navigation needs of families during this period. The provided information has to be objective, clear and sufficient for an informed decision process and should be embedded in a kind, sensitive, respectful and empathic way of communication [151,156,157,158], including emotional support [156]. Despite the importance of a timely hearing amplification, the decision process itself should not be rushed, so that parents can take the necessary time for their informed decision. 

Furthermore, affected children’s hearing device compliance may vary and the required sensitive, but persistent, educational support from parents can be challenging. Especially in the first years, family-centred support has the power to increase children’s audibility by increasing hearing device compliance [147,159,160].

When a family is faced with the decision of whether to choose a cochlear implant, the timing of this procedure is important, with respect to its possible role as stressor, potentially influencing parent–child interaction [161]. Especially in phases of insufficiency of hearing, hearing aid fitting and/or planning of cochlear implantation adds to increased parent stress [74,162,163]. 

Abu Bakar et al. (2010) [161] demonstrated that for children with cochlear implants, increased audibility does not solely account for an increase in maternal sensitivity 9 months after cochlear implantation, but that the continuous involvement in early family-centred intervention, focusing on parent–child interaction, may be an important mediator. 

In general, these data point towards the importance of a consistent family-centred support, including medical professionals, in the context of hearing device use, to increase hearing device compliance, self-efficacy regarding device handling and surveillance, and to reduce constant family irritation and stress and positively impact on parent–child interaction.

##### Diagnostic and Genetic Counselling

During the early diagnostic phase, families frequently raise the question of the cause of their child’s HL. There are multiple reasons for congenital hearing impairment, up to 70% are genetic [164]. Genetic testing, especially exome sequencing, can be used to identify the genetic cause of HL, with a diagnostic yield of 50–60% [165,166]. Genetic sensorineural HL is classically divided into a syndromic (≈33%) and non-syndromic form [164]. Due to the fast development of DNA sequencing technology, a subcategory of so-called non-syndromic mimics has arisen, presenting initially as isolated HL, but later on, additional phenotypes become evident (e.g., Usher Syndrome, Jervell-Lange-Nielson syndrome) [167]. Early diagnosis of these conditions may be beneficial in the near future, as especially for Usher syndrome, new therapeutic options are on the horizon [168]. In addition, prognosis of a possible HL progression and providing recurrence chance estimates for families make genetic testing and proper genetic counselling necessary for patients with HL [167]. Nevertheless, there exists no evidence for the optimal timepoint for genetic testing. There are also possible disadvantages of genetic testing, especially when offered early after HL diagnosis. Parents may still struggle to cope with the recent HL diagnosis and the family system may be irritated and stressed [169]. In addition, genetic testing results may also be potentially harmful, depending on the characteristics and roles of the genes involved, the family situation and clinical relevance of the genetic factors. 

The diagnosis of currently untreatable non-syndromic mimics, such as Usher syndrome, especially when a variant of unclear significance is present, may impact families’ coping mechanisms, increase stress and anxiety, intensify grief and frustration and, thus, impact parent–child interaction. 

Thus, the optimal timing to offer genetic testing for HL depends on the clinical presentation of the child and on the family and their actual circumstances. A family-adapted etiologic diagnostic process, coordinated via the families “medical home”, including guiding medical professionals from paediatrics, closely connected to clinical genetics, may best meet these needs.

##### Effect on Child Physical Health (3c)

Children’s developmental outcomes in general, including cognition, language and social-emotional development, have been linked to children’s physical health during the first years of life [170,171]. In this context, the family is known as a health promotion setting for children, whereby the child itself is an active agent in promoting their own family’s health [172]. 

Children with long-term physical conditions meet diagnostic criteria for at least one mental health disorder in up to 40% of cases [173,174]. From children with rare disease, the impact of physical health on family stress and well-being is well known, potentially impacting parent–child interaction [175,176]. 

Chronic health conditions, associated with congenital HL, such as neuro-motor impairments, swallowing and nutritional issues, visual impairments, chronic inflammatory diseases, epilepsy, neurodegenerative diseases, etc., may greatly affect parent–child interaction via a multitude of mechanisms. Chronic otitis media with effusion, for instance, not timely recognised and treated, affects audibility and, thus, parent–child interaction [177,178]. Unrecognised visual impairment directly affects parent–child interaction [179]. Untreated epileptic seizures may impact cognitive and language development and affect parent–child interaction [180]. Thus, it is obvious that, in the context of children with HL, and especially in the group of children with additional disabilities, the physical health status of the child does matter in the context of FCEI. 

##### Case Management of Children with Additional/Multiple Disabilities

Up to 40% of children with HL show additional disabilities, including autism spectrum disorder and up to 33% present with often complex, rare, or even ultra-rare syndromic forms, including progressive neurodegenerative diseases [4,5]. A systematic review by Boettcher et al. (2021) [181], on the effect of rare diseases on parents’ quality of life, showed that although disease severity is an important quality of life predictor, psychosocial factors are even more reliable predictors for parental quality of life. The increased need for care and the complex parental burden lead to limited social contacts of the family and reduced family interaction and, finally, reduced quality of life for parents, highlighting the need for family-adjusted support and navigation. Parents, especially mothers of children with complex medical needs, are more likely to suffer from depression and anxiety-related illnesses [182], and parental distress was shown to negatively impact on a child’s health [64]. For parents of developmentally disabled children, multiple behavioural and medical factors are associated with heightened parental stress [183]. Thus, the number and type of additional disabilities experienced by a child with HL may also impact their development and mental health [112,113,184]. Parents of children with disabilities have indicated that professionals provide an important source of support [185]. For parents, professionals have the responsibility to aid parents in gaining confidence and have an influential and important role in providing support to parents of children with disability [62,185].

##### Developmental Surveillance

Regular multi-professional monitoring and assessment of child and family outcomes is another principle of family-centred early intervention. The 2013 FCEI best practice guidelines highlight the importance of routine and standardised evaluation of the individual child’s development, as well as family satisfaction, self-efficacy, and well-being [31].

The inclusion of parental reports, based on long-time observations of their child in natural situations, and observations of early interventionists in the assessment, is regarded as essential. Their observations are supplemented by results of clinical examinations and standardised testing, by professionals from multiple disciplines (paediatrics, speech-language, psychology, audiology and otorhinolaryngology). Shared decision making with the families and early interventionists helps to avoid irritation and to support parents in their daily interactions with their child. 

### 3.4. Influences on Family Quality of Life (4)

Although there is robust evidence that family systems interventions do not only increase parental self-efficacy, but also parental well-being [36,37], there are still a lack of longitudinal studies on FCEI programmes for families with children with HL, which assessed family well-being as an outcome dimension. Persisting difficulties of functional communication and behaviour problems are well documented as stressors on family, therefore, an influence of child developmental outcomes on family quality of life is assumed. Dimensions, such as family happiness, family quality of life, enjoyment of the child and optimism, require further, in-depth investigation. Parent-driven movements like “Fostering Joy” [186] are promising, but need long-term evaluation. 

## 4. Implications for Practice and Research

Our model on influences of family-centred early intervention illustrates the key role of parent–child interaction, for many dimensions of child development, far beyond language. We postulate that the effects of responsive and well-adjusted parent–child interaction on child social, cognitive and mental health outcomes, are mediated by social communication development that closely interacts with language acquisition. 

Effective Early Intervention includes early identification and multi-disciplinary diagnostics, provision with hearing technology and enrolment in a family-centred early intervention programme that is closely connected with continuing multi-disciplinary surveillance and medical care (medical home). 

Specialised pathways of identification, i.e., tracking of children who failed newborn hearing screening (from birth units via otorhinolaryngological and audiological diagnostics to enrolment in early intervention programmes), that follow mandatory follow-up protocols, are required for timely access to early intervention. 

Due to the specific needs of parents of children with HL, particularly related to communication and language, hearing technology and psycho-social development, early intervention programmes, specialised for children with HL, are required. Specialisation of early intervention services also facilitates networking with other professionals in the field that is considered as mandatory. The goal of family-centred early intervention is to strengthen the functioning of the family through social-emotional support, related to family stressors, such as reaction to a child’s HL, and by capacity-building practices that focus on the quality and quantity of parent–child interaction. 

Similarly, specialised tertiary multi-disciplinary clinical services are essential for the implementation of the model. Networks of medical and non-medical professionals are required to provide high-quality intervention and avoid the irritation of families, which can be detrimental to family well-being and family communication. Regular interdisciplinary case discussions of early intervention providers and clinicians are encouraged, online communication tools can facilitate their implementation.

Staff in every discipline require training that goes beyond child development issues, focusing on partnership-based collaboration with parents that recognises family strengths, goals and priorities, and supports active family participation in making informed choices, as well as support to act on those shared decisions.

Programme monitoring to guarantee family-centredness of intervention, by use of parent feedback mechanism, beyond measures of satisfaction, use of fidelity measures by professionals, peer evaluation within professional networks and continuous assessment of multi-dimensional child and family outcomes, are deemed necessary. 

In addition to child language, psychosocial and cognitive outcomes, developmental monitoring needs to include child social communication skills, quality of parent–child interaction, family coping and quality of life. 

Despite research advancements in the field of early intervention for children with HL, evidence on effects of specific intervention measures on child and family outcomes is still scarce. Longitudinal research, including intervention measures, family behaviours and multi-dimensional child (including socio-emotional aspects) and family outcomes is required. Epidemiological studies that represent the whole variety of children (e.g., with additional disabilities, minimal hearing loss) and their families (e.g., inclusion of parents with HL, migrant background), with sufficient sample size (e.g., multi-centre studies) and multidisciplinary participation, including family representatives, are encouraged, globally, to fill the research gap.

## Data Availability

Not applicable.
